# Efficacy and safety of oral sulfate solution versus polyethylene glycol for colonoscopy: A systematic review and meta‐analysis of randomized controlled trials

**DOI:** 10.1002/deo2.70113

**Published:** 2025-04-16

**Authors:** Umar Akram, Shahzaib Ahmed, Eeshal Fatima, Eeman Ahmad, Hamza Ashraf, Syed Adeel Hassan, Zaheer Qureshi, Faryal Altaf, Daniel Buckles, Javed Iqbal, Khabab Abbasher Hussien Mohamed Ahmed

**Affiliations:** ^1^ Department of Medicine Allama Iqbal Medical College Lahore Pakistan; ^2^ Department of Medicine Fatima Memorial Hospital College of Medicine and Dentistry Lahore Pakistan; ^3^ Department of Medicine Services Institute of Medical Sciences Lahore Pakistan; ^4^ Division of Digestive Diseases and Nutrition University of Kentucky Lexington USA; ^5^ The Frank H. Netter M.D. School of Medicine at Quinnipiac University Bridgeport USA; ^6^ Department of Internal Medicine Icahn School of Medicine at Mount Sinai/BronxCare Health System New York USA; ^7^ Division of Gastroenterology and Hepatology The University of Kansas Medical Center Kansas City USA; ^8^ Nursing Department Hamad Medical Corporation Doha Qatar; ^9^ Faculty of Medicine University of Khartoum Khartoum Sudan

**Keywords:** adenoma, colonoscopy, colorectal neoplasms, meta‐analysis, polyethylene glycols

## Abstract

**Background:**

Colonoscopy is the gold standard for early detection and monitoring of colorectal cancer. Procedural effectiveness is dependent on optimal bowel preparation. Traditional polyethylene glycol (PEG) solutions are difficult to tolerate, whereas newer low‐volume alternatives, including PEG with ascorbic acid and oral sulfate solutions (OSS), offer improved efficacy and tolerability. The meta‐analysis was performed to evaluate the efficacy and safety of OSS compared to PEG for bowel preparation in colonoscopy.

**Methods:**

Studies were identified by searching PubMed, Embase, Cochrane CENTRAL, and clinicaltrials.gov from inception until June 2024. Only randomized controlled trials comparing OSS with PEG were included. Data was analyzed using R version 4.4.0 using a random effects model to calculate risk ratios (RRs) and mean differences (MDs) with 95% confidence intervals (CIs).

**Results:**

Twenty‐one studies with 6346 participants met the inclusion criteria. OSS significantly improved adenoma detection (RR, 1.13; 95% CI, 1.04–1.22; *p*‐value <0.01; I^2^ = 0%) and polyp detection rates (RR, 1.16; 95% CI, 1.06–1.26; *p*‐value <0.01; I^2^ = 0%), and had a higher Boston Bowel Preparation Scale (BBPS) score (MD, 0.31; 95% CI, 0.13–0.50; *p*‐value <0.01; I^2^ = 81%). PEG was associated with more sleep disturbances (RR, 0.45; 95% CI, 0.25–0.82; *p*‐value = 0.03; I^2^ = 0%). However, other adverse effects were similar between both solutions.

**Conclusion:**

OSS demonstrated superior adenoma and polyp detection rates. When compared to PEG, patients utilizing OSS achieved higher BBPS scores. Data gleaned support enhanced cleansing efficacy and safety of OSS as a bowel preparation regimen.

## INTRODUCTION

Colorectal cancer (CRC) is the third most diagnosed cancer worldwide, with 1.9 million new cases and 930,000 deaths estimated around the world in 2020.^1^ The incidence is projected to increase to 3.2 million new cases and 1.6 million deaths worldwide by 2040.^1^ In the US, 5.53 million cases of CRC have been reported from 1999 to 2020.^2^ Even though the incidence increases rapidly with age,^3^ the rates amongst younger adults have significantly increased.^2^ This is attributed to the introduction of screening in older adults.^3^ From 2015 to 2019, men have been observed to have a 33% higher incidence rate than women in the US,^3^ although the incidence in both sexes has decreased at the same rate over the past three decades.^2^

Several screening tests exist for CRC, including stool‐based tests such as the guaiac fecal occult blood test, fecal immunochemical test, and multi‐target stool DNA test.^4^, ^5^, ^6^, ^7^, ^8^, ^9^, ^10^, ^11^, ^12^, ^13^, ^14^ Other modalities include flexible sigmoidoscopy and colonoscopy, the most widely used method in the US.^15^ Optimal procedural yield relies on multiple factors, with standardized guidelines emphasizing the assessment of bowel preparation quality.^16^, ^17^ Adequate bowel cleansing is critical for accurate diagnosis and minimizing the risk of interval CRC.^18^, ^19^ Factors influencing bowel preparation quality include the type of preparation, split‐dose regimen, pre‐procedural diet, comorbidities, medication use, and age.^19^ Suboptimal preparation can prolong procedure time and increase the risk of complications.^20^

As bowel purgative choice is a modifiable factor, identifying the most effective and safest solution is imperative. The US Multi‐Society Task Force and the European Society of Gastrointestinal Endoscopy recommend polyethylene glycol (PEG)‐based regimens due to their proven efficacy.^20^, ^21^ Although PEG is the most commonly prescribed laxative, its large required volumes often reduce palatability and compliance.^22^, ^23^ Consequently, alternatives such as oral sulfate solution (OSS) are being investigated for colonoscopy preparation.^23^, ^24^, ^25^, ^26^, ^27^, ^28^, ^29^, ^30^, ^31^, ^32^, ^33^, ^34^, ^35^, ^36^, ^37^, ^38^, ^39^, ^40^, ^41^, ^42^, ^43^

Recent meta‐analyses by Chen et al.^44^ and Liu et al. ^45^ delineated the superior efficacy of OSS when compared to PEG for bowel preparation. This was adjudicated due to increased polyp and adenoma detection rates with OSS use. However, Chen et al.^44^ included only eight clinical trials, whereas Liu et al.^45^ included only fourteen clinical trials. Several new trials have been published to date. This systematic review and meta‐analysis aims to present the most up‐to‐date results by synthesizing data from 21 clinical trials.

## METHODS

This systematic review and meta‐analysis was conducted according to the guidelines provided by the Cochrane Handbook for Systematic Reviews of Interventions and Preferred Reporting Items for Systematic Reviews and Meta‐Analysis.^46^ This review was registered with PROSPERO, CRD42024556508. Ethical approval was not required because the study utilized already published publicly available data.

### Data sources and search strategy

We searched three electronic databases—MEDLINE (via PubMed), EMBASE (via Ovid), and Cochrane CENTRAL Library—from inception till June 2024. The clinical trial registration database (clinicaltrials.gov) was also searched for relevant trials. Reference lists in review articles were identified during this search and the final included articles were checked to identify additional potentially eligible studies. The search strategy was developed using the following MeSH keywords: “Polyethylene Glycol”, “Polyethylene ”, “colonoscopy”, and “sulfates”. Detailed search strategies are summarized in Table S1.

### Study selection

Two investigators independently assessed all potentially relevant studies. Only randomized controlled trials (RCTs) comparing OSS and PEG‐based solutions for bowel preparation during colonoscopy and reporting polyp detection rate (PDR), adenoma detection rate (ADR), cecal insertion time (CIT), cecal intubation rate (CIR), and bowel preparation scores were included. Only studies published in the English language were considered. Bowel preparation scores were assessed using the Ottawa Bowel Preparation Scale (OBPS) or the Boston Bowel Preparation Scale (BBPS). Review articles, commentaries, conference abstracts, studies lacking sufficient data, animal studies, and non‐randomized trials were excluded. Any discrepancy between the two investigators was resolved by consulting a third investigator.

### Data extraction

Two investigators independently extracted the following data from finalized studies: first author's surname, year of publication, study location, type of colonoscopy, clinical setting of colonoscopy, clinical indication, type of intervention, preparation regimen, sample size, mean age, body mass index (BMI), PDR, ADR, CIT, CIR, BBPS score, OBPS score, and adverse events. Any discrepancy between the two investigators was resolved by consulting a third investigator.

Following are the primary outcomes; ADR, PDR, and BBPS score. CIT, CIR, OBPS score, and adverse events are the secondary outcomes.

### Quality assessment

Two reviewers independently used version 2 of the Cochrane risk‐of‐bias tool for randomized trials (RoB 2) to assess the quality of the included studies. This tool evaluates five domains of a randomized study to reach an overall risk of bias judgment: bias in the randomization process, bias due to deviations from the intended interventions, bias due to missing outcome data, bias in the measurement of the outcome, and bias due to selective reporting of results. Disagreements were resolved by a third investigator.

### Statistical analysis

The statistical analysis was conducted on R version 4.4.0 using “meta” and “metasens” packages. For continuous outcomes including OBPS score, BBPS score, and CIT, mean differences (MDs) with 95% confidence intervals (CIs) were calculated. For dichotomous outcomes, ADR, PDR, CIR, and adverse events, risk ratios (RRs) with 95% CIs were calculated. The RRs with 95% CIs were pooled using the Mantel‐Haenszel^47^ method in a random effects model. The MDs were pooled using the inverse variance method. Knapp‐Hartung adjustment was applied to the CIs to account for any statistical heterogeneity.^48^ The variance was calculated using the Paule‐Mandel estimator^49^ and the restricted maximum likelihood estimator^50^ for dichotomous and continuous outcomes respectively. Heterogeneity was assessed using the cutoff values in accordance with the Cochrane Handbook of Systematic Reviews of Interventions for the Higgins I^2^ statistic, keeping in view the results of the Chi‐square test – 0%–40%: low heterogeneity; 30%–60%: moderate heterogeneity; 50%–90%: substantial heterogeneity; and 75%–100%: considerable heterogeneity.^51^ We assessed publication bias using funnel plots and Egger's test^52^ when more than ten studies were present. When less than 10 studies were present for an outcome, Doi plots and the Luis‐Furuya Kanamori index^53^ were utilized to address publication bias. Wherever more than two studies were included, we conducted a sensitivity analysis by omitting one study at a time. A two‐tailed *p*‐value <0.05 was considered statistically significant in all instances. Subgroup analysis was performed for ADR, PDR, and BBPS scores by stratifying on the basis of outpatients, morning colonoscopy, 2‐L bowel preparation protocols, mean age >55 years, Asian studies, and BMI <25 kg/m^2^. To avoid unit‐of‐analysis errors, when multiple arms of the same study were compared to the same control group, we combined the multiple intervention groups.^51^

### Certainty of evidence

We used the Grading of Recommendations Development, Assessment, and Evaluation (GRADE) approach to determine the certainty of evidence.^54^ The summary of the effects table was generated via the GRADEpro Guideline Development Tool (Table S2).^55^

## RESULTS

### Characteristics of Included Studies

**FIGURE 1:**
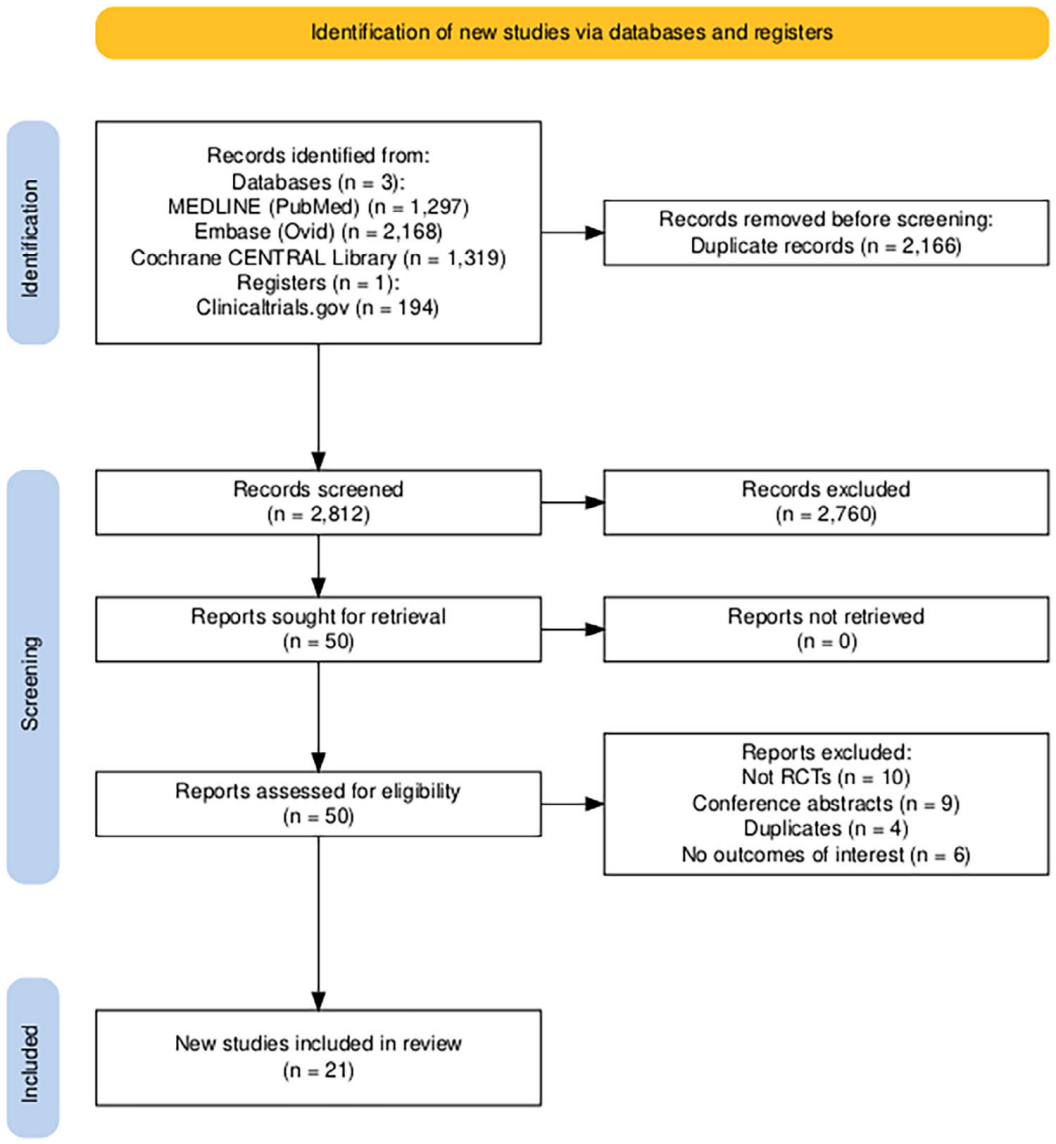
Preferred Reporting Items for Systematic Reviews and Meta‐Analysis (PRISMA) flow diagram summarizing the screening process.

A total of 21 RCTs ^23^, ^24^, ^25^, ^26^, ^27^, ^28^, ^29^, ^30^, ^31^, ^32^, ^33^, ^34^, ^35^, ^36^, ^37^, ^38^, ^39^, ^40^, ^41^, ^42^, ^43^ were included in this systematic review and meta‐analysis (Figure 1). Initial search after removing duplicates reported 2812 studies out of which 50 remained for further screening. Upon assessing their eligibility based on study design and outcomes of interest, 21 studies were included in the review. These 21 studies included a total of 6346 participants, out of which 3163 and 3183 received OSS and PEG, respectively. The mean age of participants in the PEG group was 55.9 ± 14.1 years, while that in the OSS group was 55.6 ± 13.8 years. The percentage of the male population across the studies was 56.74%. Table 1 provides detailed characteristics of the included studies and patient demographics.

**TABLE 1:** Baseline characteristics of the included studies in the systematic review and meta‐analysis.

					Interventions			Sample size		Age in years (mean ± SD)		Sex (M/F)		BMI in kg/m^2^ (mean ± SD)	
Study	Location	Type	Setting	Indicators	OSS	PEG	Regimen	OSS	PEG	OSS	PEG	OSS	PEG	OSS	PEG
Kim et al. (2017)	Korea	Morning colonoscopy	Outpatient	Screening surveillance	2L OSS	2L PEG+ASC	Split dose	83	84	55.7 ± 12.2	57.1 ± 10.0	48/35	46/38	23.8 ± 3.0	23.9 ± 2.7
Lee et al. (2019)	Korea	Morning and afternoon colonoscopy	Outpatient	Screening surveillance diagnostic treatment	2L OSS	2L PEG+ASC	Split dose	93	94	60.3 ± 11.1	60.0 ± 11.5	44/49	50/44	23.7 ± 3.4	23.3 ± 2.8
Nam et al. (2021)	South Korea	Morning colonoscopy	Outpatient	Screening surveillance diagnostic treatment	2L OSS	2L PEG+ASC	Split dose	95	94	70.92 ± 4.09	72.04 ± 4.14	48/47	68/26	NR	NR
Shah et al. (2019)	India	NR	Outpatient	Screening surveillance diagnostic treatment	1L OSS	2L PEG+ASC	Split dose	178	222	43.89 ± 13.67	43.87 ± 13. 52	112/66	148/74	NR	NR
Yang et al. (2017)	Korea	Morning colonoscopy	Outpatient	Screening diagnostic treatment	2L OSS	4L PEG+ASC	Split dose	99	100	51.2 ± 9.3	53.4 ± 8.5	53/46	63/37	23.7 ± 2.8	24.0 ± 3.0
DeMicco et al. (2018)	USA	NR	Outpatient and inpatient	Screening surveillance diagnostic	2L OSS	1L PEG+ASC	Split dose	280	276	56.8 ± 10.4	57.5 ± 10.3	156/124	141/135	29.8 ± 6.19	29.5 ± 5.62
Kwak et al. (2019)	Korea	NR	NR	Screening surveillance diagnostic	2L OSS	4L PEG+ASC	Split dose	97	96	68.6 ± 2.9	69.3 ± 2.9	43/54	46/50	NR	NR
Kwon et al. (2020)	Korea	NR	NR	Screening	2L OSS	2L PEG+ASC	Split dose	86	87	53.57 ± 10.99	56.22 ± 10.54	37/49	39/48	23.70 ± 3.00	23.90 ± 3.77
Ge et al. (2023)	China	Morning, noon, afternoon, evening, and night colonoscopy	inpatient	Screening surveillance diagnostic	2L OSS	3 L PEG	Split‐dose	588	586	72.4 ± 8.4	71.5 ± 9.2	443/145	457/129	29.1 ± 3.5	28.2 ± 3.2
Kang et al. (2024)	South Korea	Morning colonoscopy	Outpatient	Screening surveillance diagnostic	2L OSS	2L PEG+ASC	Split‐dose	127	127	74.8 ± 3.8	74.4 ± 3.7	62/65	71/56	24.4 ± 2.8	24.1 ± 3.1
Kim et al. (2022)	Korea	NR	NR	Screening surveillance	2L OSS	2L PEG+ASC	Split‐dose	89	90	57.8 ± 10.3	58.8 ± 11.5	45/44	34/56	24.0 ± 2.9	23.3 ± 3.0
Kim KO et al. (2022)	Korea	Morning colonoscopy	Outpatient	surveillance	2L OSS	2L PEG+ASC	Split‐dose	55	52	44.4 ± 17.1	41.9 ± 14.9	33/22	40/12	23.4 ± 3.0	24.0 ± 9.5
Lee et al. (2023)	Korea	Morning colonoscopy	outpatients	screening	3L OSS	2L PEG+ASC	Split‐dose	92	93	47.9 ± 14.7	48.9 ± 15.0	43/49	57/36	23.3 ± 3.0	23.6 ± 3.1
Lim et al. (2023)	Korea	Morning colonoscopy	outpatients	Screening surveillance	2L OSS	1L PEG+ASC	Split‐dose	52	52	70.5 ± 5.3	70.5 ± 4.5	31/21	30/22	24.6 ± 3.9	23.3 ± 3.2
Pan et al. (2023)	China	NR	outpatients	Screening surveillance diagnostic	3L OSS	3L PEG+ASC	Split‐dose	171	173	44.85 ± 13.32	46.59 ± 12.77	77/94	86/87	NR	NR
Socha et al. (2023)	Europe	NR	inpatient	Screening surveillance diagnostic treatment	2L OSS	70 mL/kg. PEG+ASC	Split‐dose	125	116	15.1 ± 1.6	15.3 ± 1.6	65/60	69/47	21.4 ± 4.3	21.6 ± 4.1
Woo et al. (2022)	South Korea	NR	outpatients	Screening surveillance diagnostic treatment	3L OSS	1L PEG+ASC	Split‐dose	87	85	57 (25–71)^†^	56 (20–71)^†^	46/41	53/32	NR	NR
Bhandari et al. (2023)	USA	Morning or noon colonoscopy	outpatients	Screening surveillance diagnostic	3L OSS	2L PEG	Split‐dose	250	250	56.1 ± 11.7	56.4 ± 11.8	98/152	108/142	NR	NR
Saito et al. (2021)	Japan	NR	NR	Screening surveillance diagnostic treatment	3L OSS	2L PEG+ASC	Split‐dose	202	200	53.7 ± 13.5	53.3 ± 13.3	115/87	119/81	NR	NR
							Same‐day dose	200		53.5 ± 13.6		115/85		NR	
Palma et al. (2021)	USA	NR	outpatients	Screening surveillance diagnostic	3L OSS	2L PEG+ASC	Split‐dose	314	306	NR	NR	NR	NR	NR	NR
Kmochova et al. (2021)	Czech Republic	NR	outpatients	Screening surveillance diagnostic treatment	OSS	2L PEG+ASC	Split‐dose	110	108	57.6	57.4	54/56	55/53	NR	NR
						4L PEG			109		58.8		55/54		NR

Abbreviations: ASC, Ascorbic acid; BMI, Body mass index; F, Female; L, Liter; M, Male; OSS, Oral sulfate solution; PEG, Polyethylene glycol; SD, Standard deviation.

^†^ = Median (range).

### Risk of bias assessment and certainty of evidence

The included studies exhibited a low risk of bias and high quality when assessed using the RoB‐2 tool. Details of the quality assessment are shown in Figures S1 and S2. We evaluated the certainty of evidence under inconsistency, imprecision, indirectness, publication bias, and risk of bias. The certainty of evidence along with their details are provided in Table S2.

### Results of meta‐analysis

#### Adenoma and polyp detection rates

**FIGURE 2:**
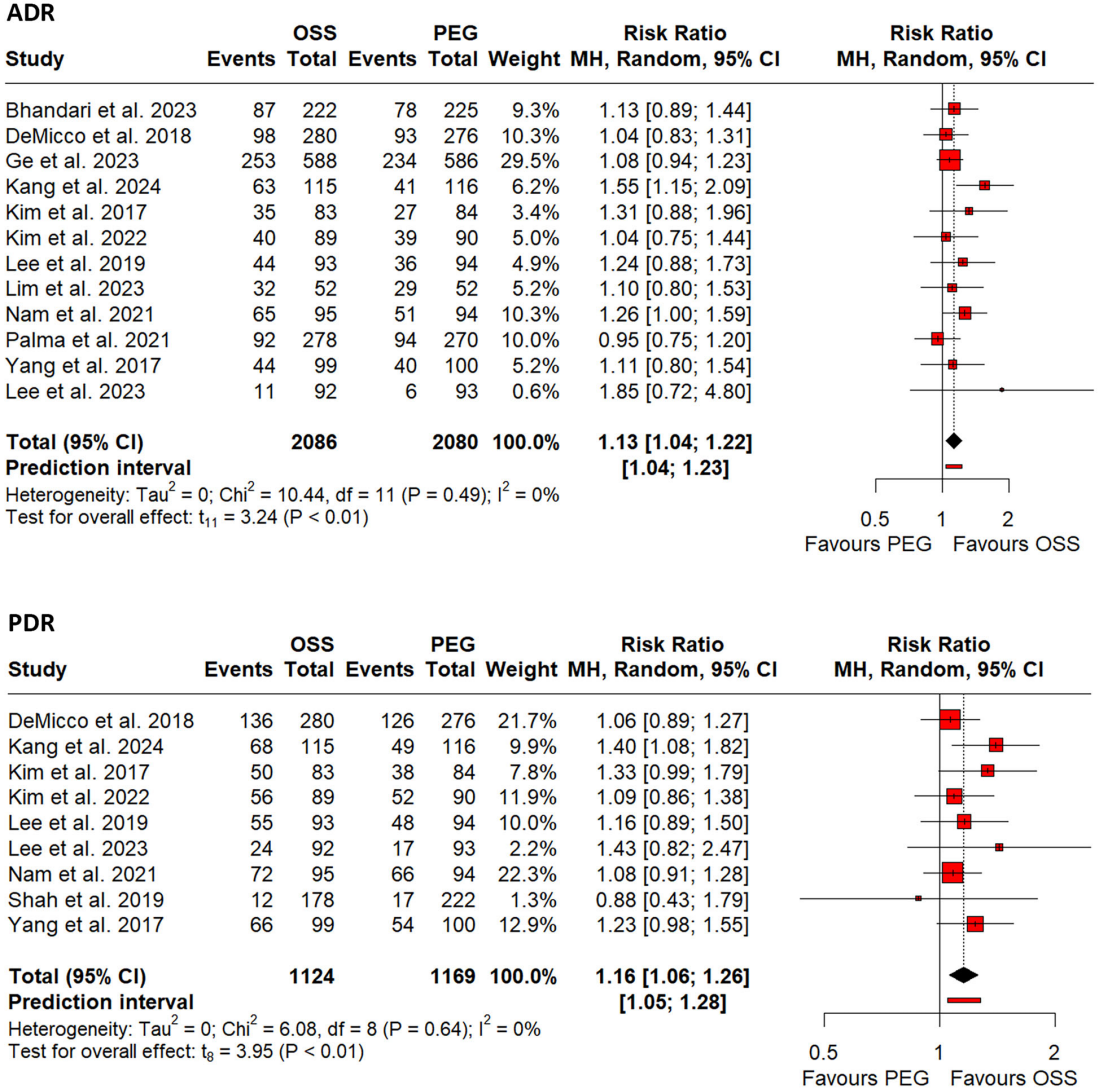
(a) Forest plot of pooled adenoma detection rate (ADR). (b) Forest plot of pooled polyp detection rate (PDR).

ADR was reported by 12 RCTs ^24^, ^26^, ^27^, ^29^, ^30^, ^35^, ^36^, ^37^, ^38^, ^40^, ^41^, ^42^ involving 4166 participants, while PDR was reported by nine RCTs ^24^, ^25^, ^26^, ^27^, ^29^, ^30^, ^37^, ^38^, ^40^ involving 2293 participants. The use of OSS was significantly associated with an increase in the rate of both adenoma (RR: 1.13, 95% CI: 1.04–1.22, *p*‐value <0.01; I^2^ = 0%, Figure 2a) and polyp (RR: 1.16, 95% CI: 1.06–1.26, *p*‐value <0.01; I^2^ = 0%, Figure 2b) detection rates. No significant changes in results were observed by omitting one study at a time for either adenoma or polyp detection rates, highlighting the robustness of the results.

**FIGURE 3:**
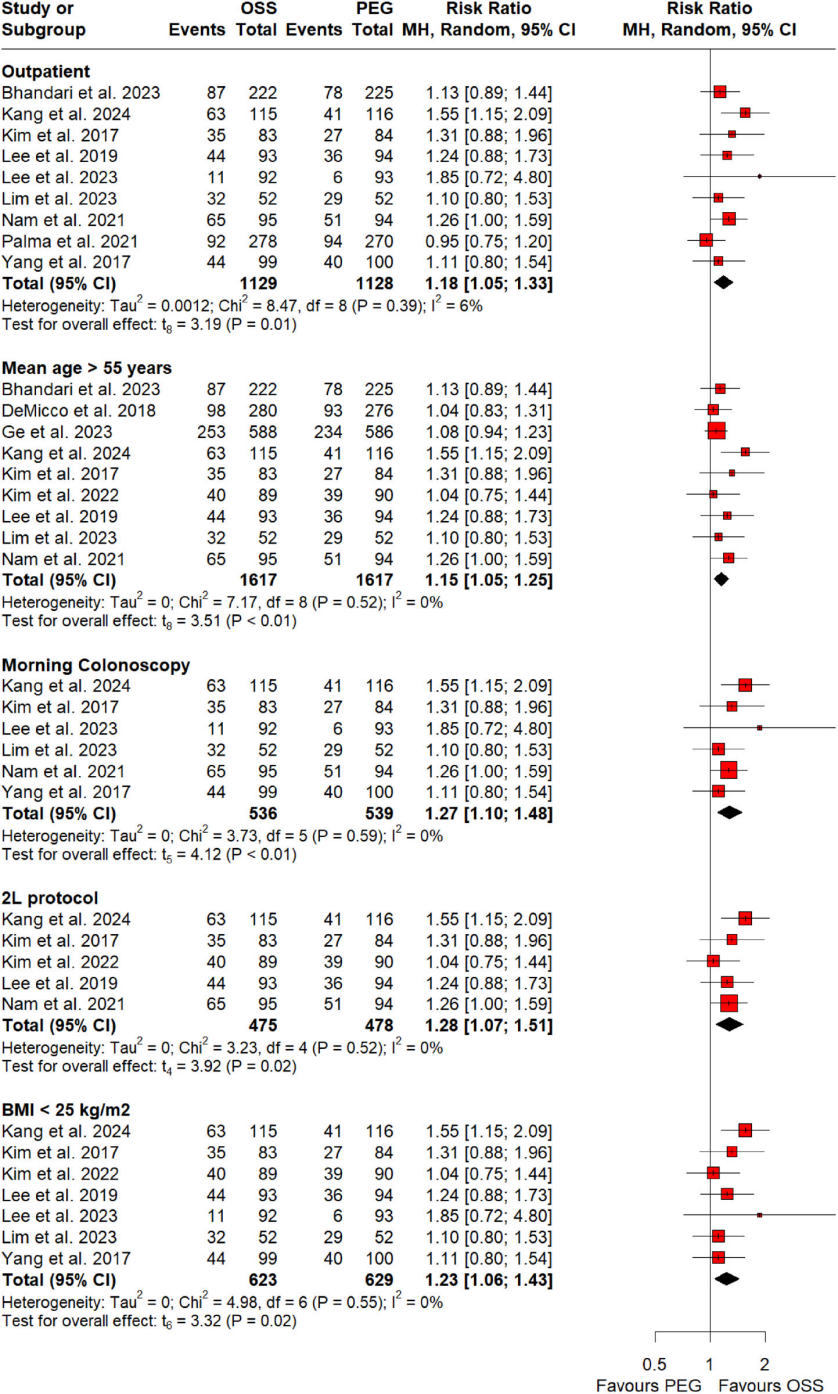
Subgroup analysis of adenoma detection rate on the basis of mean age >55 years, Morning colonoscopy, 2‐L protocol, BMI <25 kg/m^2^, and outpatient settings.

**FIGURE 4:**
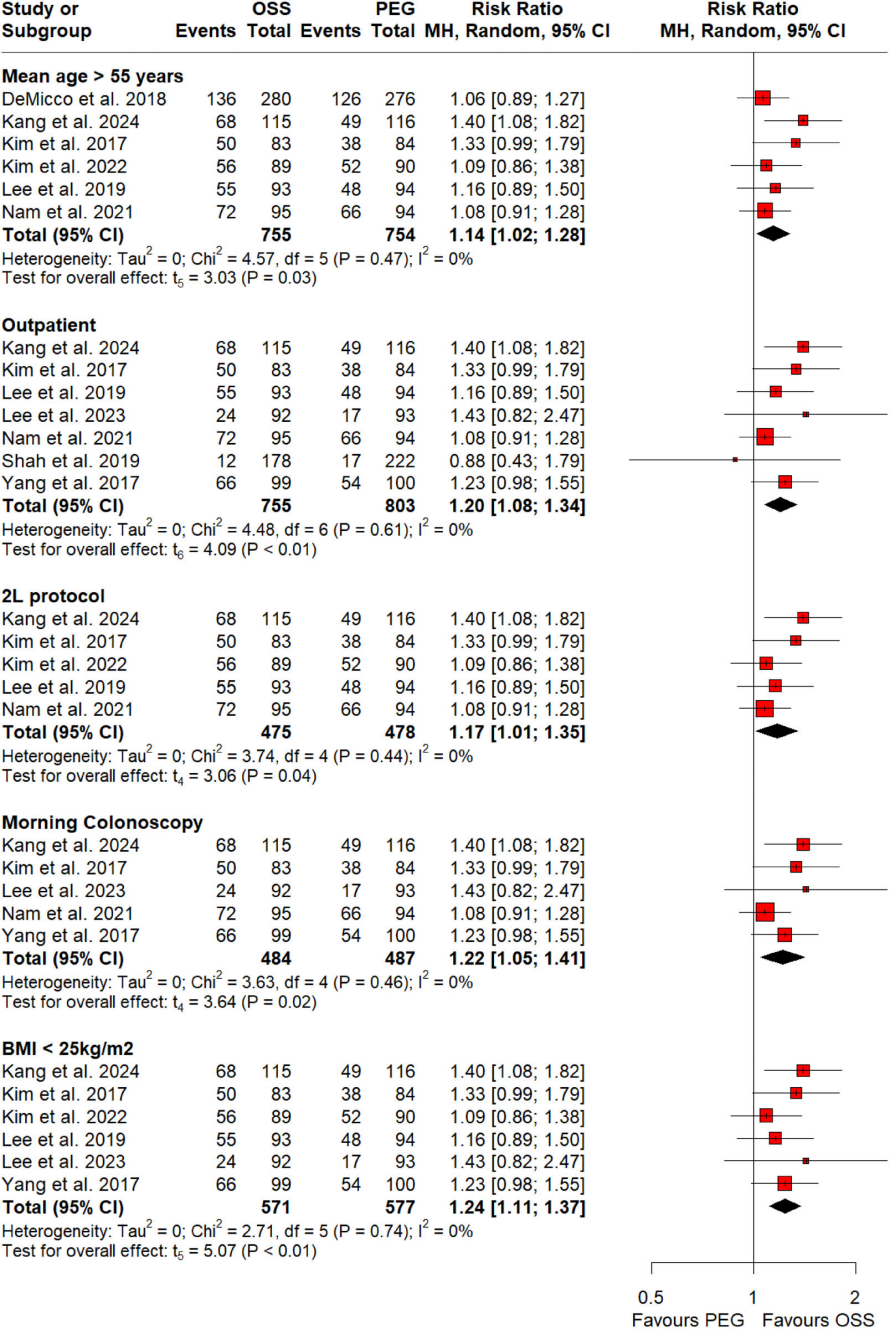
Subgroup analysis of polyp detection rate on the basis of mean age >55 years, morning colonoscopy, 2‐L protocol, BMI <25 kg/m^2^, and outpatient settings.

RCTs that were pooled in this meta‐analysis varied across different variables including mean age of patients, type of clinical setting, timing of colonoscopy, dose of bowel preparation, and BMI. A subgroup analysis was conducted to eliminate the impact of these factors on adenoma and polyp detection rates. Our subgroup analyses revealed a significant increase in PDR (Figure 3) with mean age >55 years (RR: 1.14; 95% CI: 1.02–1.28, *p*‐value = 0.03; I^2^ = 0%; six RCTs; 1509 participants), morning colonoscopy (RR: 1.22; 95% CI: 1.05–1.41, *p*‐value = 0.02; I^2^ = 0%; five RCTs; 971 participants), 2‐L protocol (RR: 1.17; 95% CI: 1.01–1.35, *p*‐value = 0.04; I^2^ = 0%; five RCTs; 953 participants), BMI <25 kg/m^2^ (RR: 1.24; 95% CI: 1.11–1.37, *p*‐value <0.01; I^2^ = 0%; six RCTs; 1148 participants), and outpatient clinical settings (RR: 1.20; 95% CI: 1.08–1.34, *p*‐value <0.01; I^2^ = 0%; seven RCTs; 1558 participants) with the use of OSS. Similar results were reported on subgroup analyses for ADR (Figure 4) with mean age >55 years (RR: 1.15; 95% CI: 1.05–1.25, *p*‐value <0.01; I^2^ = 0%; nine RCTs; 3234 participants), morning colonoscopy (RR: 1.27; 95% CI: 1.10–1.48, *p*‐value <0.01; I^2^ = 0%; six RCTs; 1075 participants), 2‐L protocol (RR: 1.28; 95% CI: 1.07–1.51, *p*‐value = 0.02; I^2^ = 0%; five RCTs; 953 participants), BMI <25 kg/m^2^ (RR: 1.23; 95% CI: 1.06–1.43, *p*‐value = 0.02; I^2^ = 0%; seven RCTs; 1252 participants), and outpatient clinical settings (RR: 1.18; 95% CI: 1.05–1.33, *p*‐value = 0.01; I^2^ = 6%; nine RCTs; 2257 participants).

#### Bowel preparation scores

**FIGURE 5:**
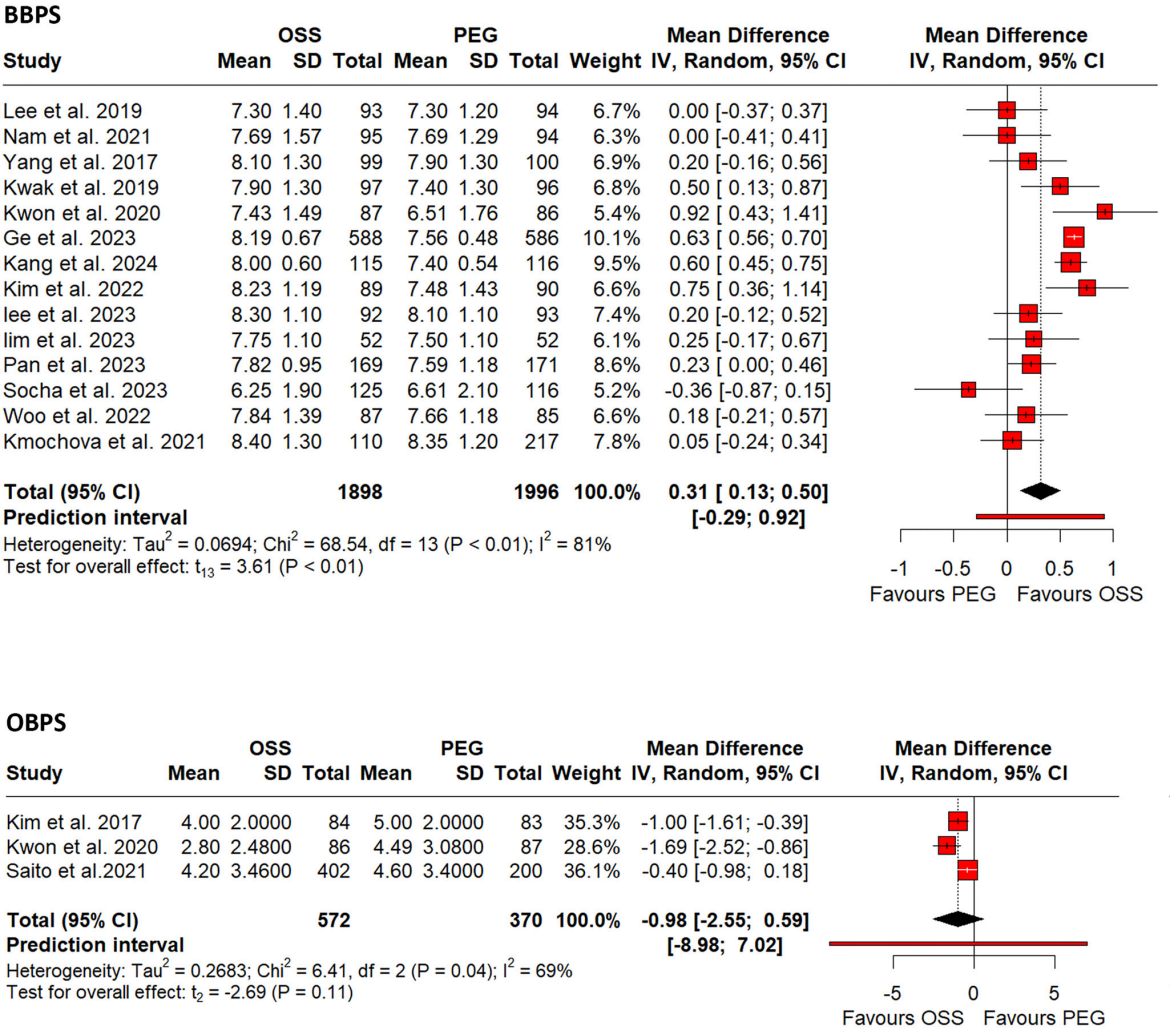
(a) Forest plot of Pooled Boston Bowel Preparation Scale (BBPS) score. (b) Forest plot of Pooled Ottawa Bowel Preparation Scale (OBPS) score.

**FIGURE 6:**
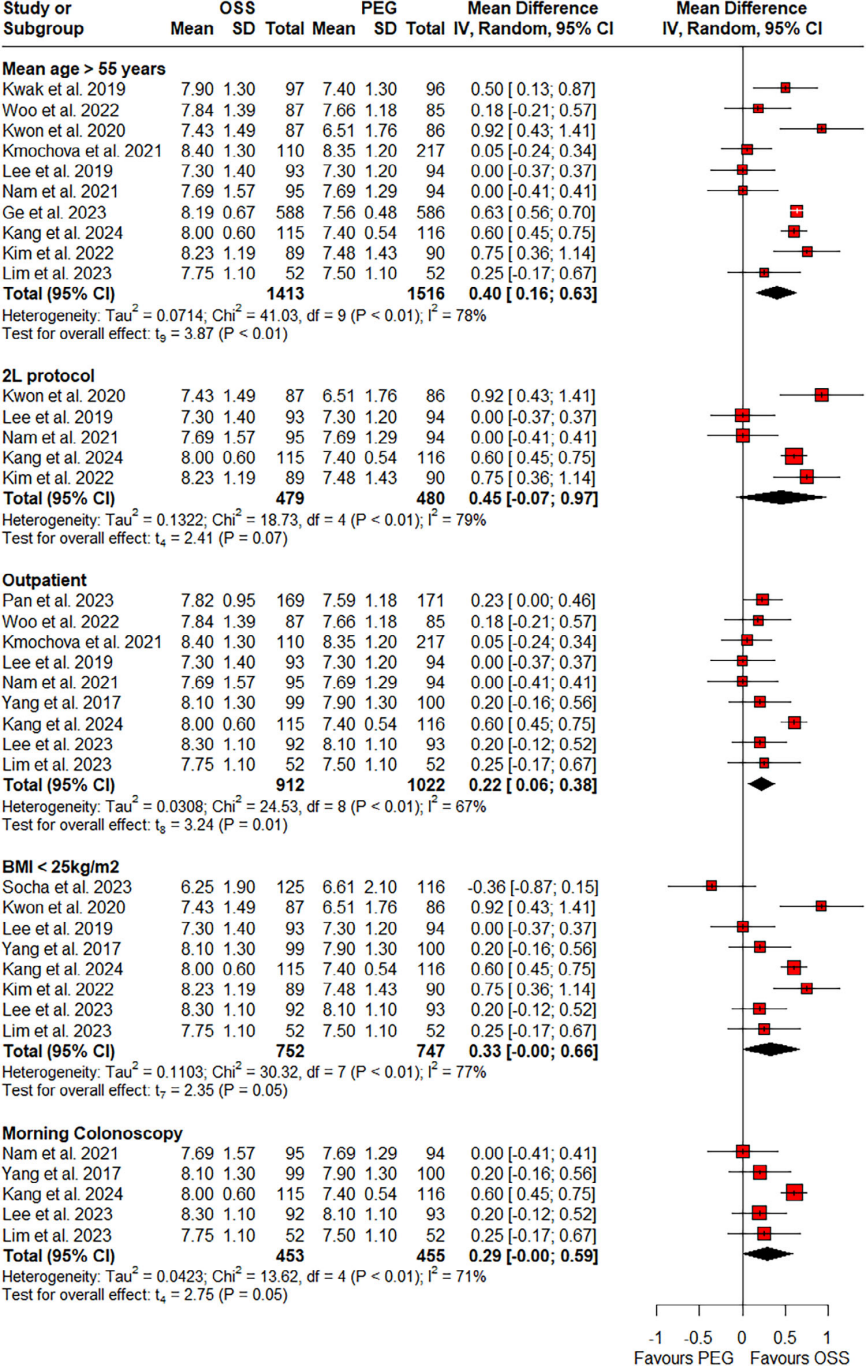
Subgroup analysis of BBPS score on the basis of mean age >55 years, morning colonoscopy, 2‐L protocol, BMI <25 kg/m^2^, and outpatient settings.

For BBPS, 14 RCTs^23^, ^24^, ^26^, ^27^, ^28^, ^31^, ^32^, ^34^, ^36^, ^37^, ^38^, ^40^, ^41^, ^43^ comprising 3894 participants were pooled. The use of OSS was associated with a significantly higher BBPS score (MD: 0.31, 95% CI: 0.13– 0.50, *p* <0.01; I^2^ = 81%, Figure 5a). Results of subgroup analyses and sensitivity analyses are provided in Heading S1 (Figure 6).

OBPS was assessed in three RCTs^28^, ^29^, ^33^ involving 942 participants. However, no significant difference in OBPS scores was observed with the use of either solution (MD: ‐0.98, 95% CI: ‐2.55–0.59, *p* = 0.11; I^2^ = 69%, Figure 5b).

#### CIT and CIR

**FIGURE 7:**
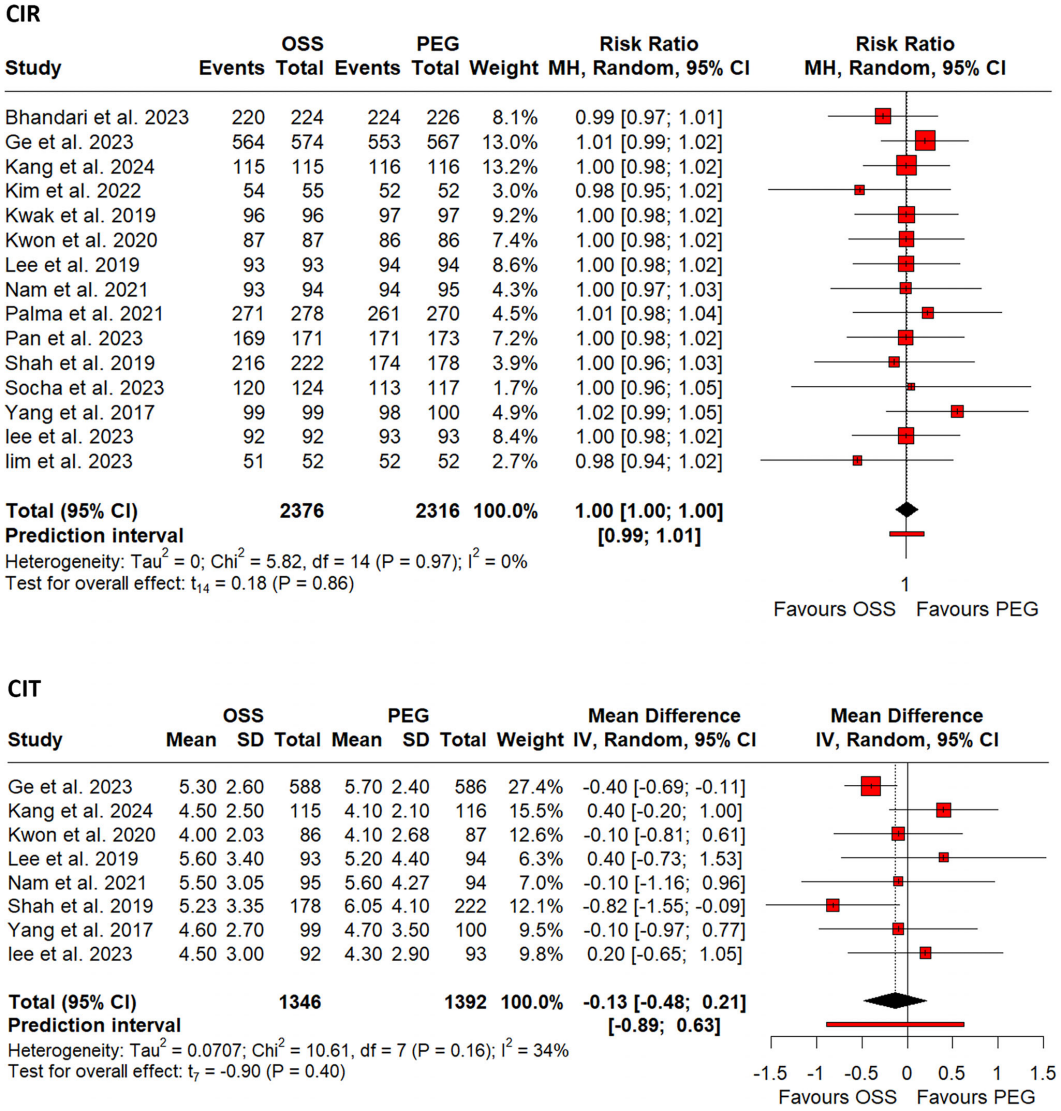
(a) Forest plot of pooled cecal insertion time (CIT). (b) Forest plot of pooled cecal intubation rate (CIR).

We pooled eight RCTs ^24^, ^25^, ^26^, ^27^, ^28^, ^37^, ^40^, ^41^ having a total of 2738 participants for CIT. Comparable results between the OSS and PEG groups were observed (MD: ‐0.13, 95% CI: ‐0.48–0.21, *p*‐value = 0.40; I^2^ = 34%, Figure 7a). Similarly, pooled analysis of 15 RCTs^23^, ^24^, ^25^, ^26^, ^27^, ^28^, ^32^, ^34^, ^35^, ^36^, ^37^, ^39^, ^40^, ^41^, ^42^ involving 4692 participants for CIR had comparable results between the two groups (RR 1.00, 95% CI: 1.00–1.00, *p*‐value = 0.86; I^2^ = 0%, Figure 7b). Omitting Kang et al.^40^ removed heterogeneity from the pooled analysis for CIT, resulting in an estimate that showed a significant increase in the CIT with the use of PEG (MD: ‐0.30, 95% CI: ‐0.58–0.02, *p*‐value = 0.04, Figure S6). No significant results were observed for the L1O analysis of CIR.

#### Adverse events

In our pooled analysis, we observed the risk of sleep disturbances to be significantly associated with the use of PEG solutions (*p*‐value = 0.03), with incidents reported in two out of 206 participants for OSS and five out of 204 participants for PEG.

The risk of gastrointestinal adverse effects was comparable between the OSS and PEG groups. Nausea was reported by 592 out of 3099 OSS participants and 597 out of 3023 PEG participants (*p* = 0.58), vomiting by 160 out of 3100 OSS participants and 133 out of 3023 PEG participants (*p* = 0.23), and abdominal pain by 151 out of 1803 OSS participants and 155 out of 1942 PEG participants (*p* = 0.51). Similarly, abdominal distension or bloating occurred in 360 out of 1748 OSS participants and 368 out of 1777 PEG participants (*p* = 0.52). Abdominal discomfort was reported by 15 out of 657 OSS participants and 36 out of 651 PEG participants (*p* = 0.84).

The results for all other adverse events were comparable between the two groups. Thirst was experienced by 70 out of 382 participants in the OSS group and 79 out of 377 in the PEG group (*p* = 0.67). Paresthesias and itching were reported in five out of 374 OSS participants and seven out of 373 PEG participants (*p* = 0.55). Numbness was observed in four participants from each group, among 232 OSS and 231 PEG participants (*p* = 0.95). Mucosal changes were reported in 14 out of 244 OSS participants and 13 out of 242 PEG participants (*p* = 0.84). Headaches were reported in 14 out of 627 participants in the OSS group and nine out of 666 participants in the PEG group (*p* = 0.56). Dizziness was experienced by 45 out of 275 OSS participants and 43 out of 273 PEG participants (*p* = 0.87). Results for pooled adverse events are given in Table 2.

**TABLE 2:** Adverse events reported by the included studies.

	No. of studies	Events/total		Pooled RR			Publication bias
Adverse event		OSS	PEG		95% CI (*p*‐value)	I^2^	
Nausea	18	592/3099	597/3023	1.06	0.86–1.30 (0.58)	61%	ET = 0.9064
Vomiting	18	160/3100	133/3023	1.26	0.85–1.88 (0.23)	54%	ET = 0.8188
Abdominal distention	14	360/1748	368/1777	0.96	0.86–1.08 (0.52)	0%	ET = 0.2302
Abdominal pain	14	151/1803	155/1942	1.09	0.82–1.45 (0.51)	12%	ET = 0.9483
Headache	4	14/627	9/666	0.72	0.14–3.75 (0.57)	16%	LI = 1.28
Thirst	5	70/382	79/377	0.86	0.33–2.21 (0.67)	71%	LI = 0.43
Dizziness	3	45/275	43/273	0.93	0.14–6.28 (0.88)	72%	LI = 0.85
Mucosal change	4	22/383	17/383	0.76	0.25–2.30 (0.40)	0%	LI = 0.29
Paresthesia	4	5/374	7/373	0.72	0.15–3.47 (0.55)	0%	LI = ‐3.13
Numbness	3	4/232	4/231	0.97	0.15–6.18 (0.95)	0%	LI = ‐0.95
Sleep disturbances	3	2/206	5/204	0.45	0.25–0.82 (0.03)^*^	0%	LI = 2.07
Abdominal discomfort	2	15/657	36/651	0.76	0.00–643036.92 (0.84)	83%	–

CI, Confidence interval; ET, Egger's test;I^2^, Heterogeneity; LI, Luis Furuya‐Kanamori Index; OSS, Oral sulfate solution; PEG, Polyethylene glycol; RR, Risk Ratio.

* indicates significant *p*‐value.

#### Subgroup analysis on Asian studies

A total of 17 RCTs (out of 21) were conducted in Asia. Subgroup analysis revealed significantly increased ADR, PDR, and BBPS scores in the OSS group when compared with the PEG group (Figure S7–S9).

### Publication bias

Detailed results of publication bias are provided in Heading S2.

## DISCUSSION

In this updated systematic review and meta‐analysis, we studied the efficacy and safety of OSS versus PEG as bowel preparation regimens for colonoscopy. Our results reported a significant improvement in the primary clinical outcomes: adenoma and polyp detection rates and BBPS. However, no significant treatment differences were observed in OBPS, CIT, and CIR. The analysis of adverse events revealed a significant association of sleep disturbances with PEG. On the other hand, thirst, paresthesias, itching, numbness, nausea, mucosal change, headache, dizziness, and gastrointestinal disturbances showed no significant association with either OSS or PEG. All the trials included in our analysis had a low or moderate risk of bias, confirming our study's robustness.

Colonoscopy is key for CRC screening and prevention, with its effectiveness relying on proper bowel preparation. Split‐dose regimens like OSS and PEG enhance detection and safety.^44^, ^56^, ^57^ A previous meta‐analysis of 10 studies conducted in 2022^44^ reported significantly higher polyp and adenoma detection rates, as well as BBPS scores with the use of OSS compared to PEG, which is consistent with our findings. However, similar to our results, no significant treatment differences were observed with respect to CIT and CIR.^44^ Another meta‐analysis by Ali et al.^57^ conducted in 2021 compared OSS versus PEG for colonoscopy preparation. They reported a higher likelihood of excellent bowel preparation with OSS than PEG, which resonates with our results. A prospective randomized study compared four different types of bowel cleansing agents for colonoscopy, which included OSS, PEG, PEG‐ascorbate, and magnesium citrate + sodium picosulfate.^43^ Their results favored excellent bowel preparations with the use of OSS and PEG. However, the difference was not statistically different from the other groups (*p* = 0.37).^43^ A recent meta‐analysis by Liu et al.,^45^ which included 14 RCTs and a total of 4526 patients, reported significant improvements in adenoma and polyp detection rates, as well as better BBPS scores with the use of OSS. These findings align with our results. However, Liu et al. also observed a significant reduction in CIT with OSS, a finding that contrasts with our study, which reported no significant treatment differences in CIT. However, the omission of Kang et al.^40^ resulted in a significant reduction in CIT with OSS and was the possible cause of the difference between our results and those of Liu et al.^45^

While colonoscopy is crucial for identifying and mitigating the risk of CRC, its efficacy may vary among different age groups. Therefore, guidelines suggest varying recommendations for different age cohorts.^58^ In the elderly population, the risk‐to‐benefit analysis of a colonoscopy drives procedural recommendations. This is due to the well‐established risk of colonoscopy‐associated complications with advancing age.^59^ A meta‐analysis concluded that octogenarians had a 70% higher chance of experiencing an adverse event.^59^ Likewise, patients under the age of 45 have a lower risk of CRC and are therefore not strongly advised to undergo screening colonoscopy.^60^ Most of our included studies are from Asia. According to a 2019 Global Burden of Disease analysis, East Asia (including China, Japan, Korea, and other countries) was the region that was the worst affected by CRC.^61^ Another study conducted in New York City reported the lowest prevalence of timely colonoscopy screening in Asian Americans from 2012 to 2018.^62^ Thus, emerging evidence suggests that variations in follow‐up colonoscopy utilization and ADRs may be key contributors to racial disparities in CRC outcomes, ultimately affecting CRC incidence, mortality, and the life‐years gained through screening.^63^ Similarly, increased BMI is associated with a higher CRC incidence and mortality.^64^ It is hypothesized that obesity may also be associated with inadequate bowel cleansing.^65^ Furthermore, substantial literature has suggested that procedural timing is an independent predictor of colonoscopy outcomes.^66^, ^67^ The colonoscopy completion rates and adequate bowel preparation rates have been reported to be lower in the afternoon scheduled procedures than in the morning procedures.^67^ In addition to the choice of bowel cleansing agent, additional considerations include the type of dosing regimen, including split or non‐split, and low‐volume or high‐volume dosing regimens. Another meta‐analysis conducted in 2020^68^ compared low‐volume split dose versus high‐volume split dose bowel cleansing regimens. Low‐volume split‐dose regimens employed OSS and PEG‐citrate, PEG‐ascorbic acid, and OSS, while high‐volume split‐dose regimens included PEG‐based regimens. Their analysis of 17 RCTs concluded that low‐volume split‐dose regimens are as effective as high‐volume regimens but with a better safety profile and preparation quality.^68^, ^69^, ^70^ This is because split‐dose and low‐volume regimens can enhance drug tolerance and patient adherence.^69^, ^70^ Additionally, a split dose option is also endorsed by the American College of Gastroenterology.^71^ A propensity‐matched analysis conducted at Massachusetts General Hospital reported that the use of low‐volume regimens could significantly decrease the time between bowel preparation and colonoscopy.^70^ To account for these variations, we also performed a subgroup analysis based on different factors such as age, BMI, volume, race, and time of colonoscopy to evaluate the treatment effects across different subpopulations in our meta‐analysis. The results of our subgroup analysis demonstrated a significant improvement in ADR and PDR with the use of OSS across all the subgroups, which include outpatient, mean age >55 years, Asian race, morning colonoscopy, 2‐L protocol (low‐volume regimen), and BMI <25 kg/m^2^. Similar findings were reported by Chen et al.^44^ However, Chen et al.^44^ did not conduct a subgroup analysis for BBPS scores, whereas our subgroup analysis for morning colonoscopy, 2‐L protocol, and BMI <25 kg/m^2^ indicated comparable results for BBPS scores, suggesting that the use of OSS in these circumstances may not have a significant improvement on bowel preparation, but may be considered due to the significant improvement in ADR and PDR.

The same propensity‐matched analysis also suggested that using low‐volume regimens also prevents unnecessary hospital bed days and potentially lowers healthcare expenses.^70^ A cost‐effective analysis conducted by Huynh et al. concluded that OSS as a cleansing agent was associated with possible cost savings as compared to PEG from a payer's perspective.^72^ This cost‐effectiveness also favors the clinical use of OSS over PEG, thus supporting our results.

Nevertheless, OSS and PEG have some side effects and complications. Gastrointestinal disturbances such as nausea, vomiting, bloating, and cramps are very common. Other adverse events include headache, fatigue, sleep disturbances, dizziness, thirst, paresthesia, itching, numbness, dehydration, etc.^25^, ^43^ We also pooled the commonly reported adverse events in the included studies to determine the safety profile of OSS and PEG. Our analysis concluded that there was no significant association between these adverse effects and either group except for sleep disturbances, which were significantly associated with PEG. A study suggested that these sleep disturbances can be prevented by administering bowel cleansing preparation early in the evening.^69^ A previous meta‐analysis by Chen et al. did not assess the safety outcomes,^44^ while the meta‐analysis by Ali et al. revealed a significantly increased risk of gastrointestinal disturbances such as nausea and vomiting with the use of oral sodium sulfate, which is in contrast with our findings.^57^ The difference in results can be attributed to several factors. Ali et al. included only studies that utilized low‐volume PEG and applied a fixed‐effects model for certain outcomes. Furthermore, their sample size and the number of included studies were significantly smaller than ours. However, similar to our investigation, Liu et al.^45^ also observed no significant differences in the incidence of adverse events between OSS and PEG, with the exception of dizziness, which was significantly associated with the OSS group. However, sleep disturbances were not assessed by Liu et al.,^45^ which has been significantly associated with the use of PEG compared to OSS in our study.

Our study has certain strengths and limitations. These have been mentioned in detail in Heading S3.

## CONCLUSION

Our data supports the superior efficacy of OSS as a bowel preparation regimen, notably enhancing adenoma and polyp detection rates while also improving bowel preparation scores. Although both OSS and PEG had an overall comparable safety profile, there were some concerns about increased sleep disturbances with PEG. Nevertheless, the superior clinical and safety benefits of OSS support its use as a suitable alternative to PEG. Thus, adopting OSS could potentially improve patient outcomes and comfort during colonoscopy.

## CONFLICT OF INTEREST STATEMENT

None.

## ETHICS STATEMENT

N/A

## Supporting information




**Table S1**: Detailed Search Strategy of Each Database
**Table S2**: GRADE Certainty of Evidence Assessment
**Figure S1**: Traffic Light Plot showing Risk of Bias Assessment
**Figure S2**: Summary Plot showing Risk of Bias Assessment
**Figure S3**: L1O Analysis for BBPS Score Morning Colonoscopy Subgroup
**Figure S4**: L1O Analysis for BBPS Score Outpatient Clinical Setting Subgroup
**Figure S5**: L1O Analysis for BBPS Score BMI < 25 kg/m2 Subgroup
**Figure S6**: L1O Analysis for Cecal Insertion Time
**Figure S7**: Subgroup Analysis of Adenoma Detection Rate (ADR) on basis of Asian Studies
**Figure S8**: Subgroup Analysis of Polyp Detection Rate (PDR) on basis of Asian Studies
**Figure S9**: Subgroup Analysis of BBPS score on basis of Asian Studies
