# Supplementary material for: Efficacy and safety of oral sulfate solution versus polyethylene glycol for colonoscopy: A systematic review and meta‐analysis of randomized controlled trials
Source: DEN Open. 2025 Apr 16;5(1):e70113. doi: 10.1002/deo2.70113 (PMC12003215; doi:10.1002/deo2.70113)

**Supplementary File**

**Efficacy and Safety of Oral Sulfate Solution versus Polyethylene Glycol for Colonoscopy: Systematic Review and Meta-analysis of Randomized Controlled Trials**

The authors created this file to provide a better understanding of their work.

**Supplementary Heading S1:** *Subgroup Analyses and Sensitivity Analysis of Boston Bowel Preparation Scale (BBPS) Score*

Subgroup analyses revealed a significantly higher BBPS score with mean age > 55 years (MD: 0.40, 95% CI: 0.16 to 0.63, p-value < 0.01; I2 = 78%; 10 RCTs; 2,929 participants) and in outpatient clinical settings (MD: 0.22, 95% CI: 0.06 to 0.38, p-value = 0.01; I2 = 67%; 9 RCTs; 1,934 participants). However, results were comparable for subgroups: 2-litre protocol (MD: 0.45, 95% CI: -0.07 to 0.97, p-value = 0.07; I2 = 79%; 5 RCTs; 959 participants), BMI < 25 kg/m2 (MD: 0.33, 95% CI: -0.00 to 0.66, p-value = 0.05; I2 = 77%; 8 RCTs; 1,499 participants), and morning colonoscopy (MD: 0.29, 95% CI: -0.00 to 0.59, p-value = 0.05; I2 = 71%; 5 RCTs; 908 participants) (***Figure 6***). Upon omitting Kang et al. 202440, the leave-one-out analysis revealed a significant decrease in heterogeneity for morning colonoscopy subgroup (MD: 0.17, 95% CI: 0.01 to 0.33, p-value = 0.04; I2 = 0%, ***Supplementary Figure S3***) and outpatient clinical setting subgroup (MD: 0.15, 95% CI: 0.07 to 0.23, p-value < 0.01; I2 = 0%, ***Supplementary Figure S4***). Upon omitting Socha et al. 202332 from BMI < 25 kg/m2 subgroup, a significantly higher score was observed for OSS, however the heterogeneity remained high (MD: 0.41, 95% CI: 0.11 to 0.71, p-value = 0.02, I2 = 71%, ***Supplementary Figure S5***).

**Supplementary Heading S2:** *Results of Publication Bias*

Publication bias was detected in BBPS (Egger’s test, p-value = 0.0073); however, no publication bias was observed in ADR (Egger’s test, p-value = 0.1205), CIR (Egger’s test, p-value = 0.3312), nausea (Egger’s test, p-value = 0.9064), vomiting (Egger’s test, p-value = 0.8188), abdominal pain (Egger’s test, p-value = 0.9483), and abdominal distention or bloating (Egger’s test, p-value = 0.2302). No asymmetry was observed for OBPS (LFK index = -0.74), dizziness (LFK index = -0.8), numbness (LFK index = 0.93), and thirst (LFK index = -0.56). CIT (LFK index = 1.17) and headache (LFK index = -1.36) had minor asymmetry. Major asymmetry was observed for PDR (LFK index = 2.28), mucosal change (LFK index = 4.23), paresthesias or itching (LFK index = 3.13), sleep disturbances (LFK index = -2.17), and abdominal discomfort (LFK index = 3.52).

**Supplementary Heading S3:** *Strengths and Limitations of this Systematic Review and Meta-analysis*

Our study has certain strengths. First, the effectiveness of OSS compared to PEG was thoroughly examined using RCTs, and the results were evaluated based on the guidelines established by the GRADE working group, which indicates a moderate level of certainty. Second, our study employed a substantial sample size and conducted a rigorous analysis to evaluate the efficacy and safety outcomes. We conducted a subgroup analysis based on possible variables that could affect the results of our meta-analysis. This includes the mean age of patients, Asian race, type of clinical setting, timing of colonoscopy, doses of bowel preparation solution, and BMI. Third, almost all the studies included in our review exhibited a low risk of bias, which adds to the robust nature of our findings. Lastly, the individual studies were conducted in different countries, warranting the generalizability of our findings. There are some limitations that need to be considered. Significant heterogeneity was observed in the analysis of certain outcomes, such as BBPS and OBPS scores, thirst, dizziness, nausea, vomiting, and abdominal discomfort. However, we addressed this issue by using the random effects model first and conducting a subgroup analysis and leave-one-out sensitivity analysis. Additionally, strong evidence of publication bias was also observed in the analysis of few outcomes, which might be due to the unpublished studies. For outcomes affected by publication bias, more robust studies are required to confirm these associations.

**Supplementary Table S1:** Detailed Search Strategy of Each Database

| **Database** | **Search Strategy** | **# of Results** |
| --- | --- | --- |
| PubMed (MEDLINE) | (Macrogol OR Macrogols OR Polyethylene Glycol OR Glycol, Polyethylene OR Glycols, Polyethylene OR Polyethyleneoxide OR Polyethyleneoxides OR Polyoxyethylene OR Polyglycol OR Polyglycols OR Polyethylene Oxide OR Oxide, Polyethylene OR Oxides, Polyethylene OR Polyethylene Oxides OR Carbowax) AND (Colonoscopies OR Colonoscopic Surgical Procedures OR Colonoscopic Surgical Procedure OR Procedure, Colonoscopic Surgical OR Procedures, Colonoscopic Surgical OR Surgical Procedure, Colonoscopic OR Colonoscopic Surgery OR Colonoscopic Surgeries OR Surgeries, Colonoscopic OR Surgery, Colonoscopic OR Surgical Procedures, Colonoscopic) | 1297 |
| Embase (Ovid) | ***Set#1***  (Polyethylene Glycols or polyethylene).mp. [mp=title, abstract, heading word, drug trade name, original title, device manufacturer, drug manufacturer, device trade name, keyword heading word, floating subheading word, candidate term word]  ***Set#2***  Colonoscopy.mp. or colonoscopy/  ***Set#3***  1 and 2 | 2168 |
| Cochrane CENTRAL | (Macrogol OR Macrogols OR Polyethylene Glycol OR Glycol, Polyethylene OR Glycols, Polyethylene OR Polyethyleneoxide OR Polyethyleneoxides OR Polyoxyethylene OR Polyglycol OR Polyglycols OR Polyethylene Oxide OR Oxide, Polyethylene OR Oxides, Polyethylene OR Polyethylene Oxides OR Carbowax) AND (Colonoscopies OR Colonoscopic Surgical Procedures OR Colonoscopic Surgical Procedure OR Procedure, Colonoscopic Surgical OR Procedures, Colonoscopic Surgical OR Surgical Procedure, Colonoscopic OR Colonoscopic Surgery OR Colonoscopic Surgeries OR Surgeries, Colonoscopic OR Surgery, Colonoscopic OR Surgical Procedures, Colonoscopic) | 1319 |
| Clinicaltrials.gov |  | 194 |

**Supplementary Table S2:** GRADE Certainty of Evidence Assessment

| **Certainty assessment** | | | | | | | **№ of patients** | | **Effect** | | **Certainty** |
| --- | --- | --- | --- | --- | --- | --- | --- | --- | --- | --- | --- |
| **№ of studies** | **Study design** | **Risk of bias** | **Inconsistency** | **Indirectness** | **Imprecision** | **Other considerations** | **Oral sulfate solution** | **Polyethylene glycol** | **Relative  (95% CI)** | **Absolute  (95% CI)** |  |
| **Adenoma detection rate** | | | | | | | | | | | |
| 12 | randomised trials | not serious | not serious | not serious | not serious | none | 864/2086 (41.4%) | 768/2080 (36.9%) | **RR 1.13**  (1.04 to 1.22) | **48 more per 1,000**  (from 15 more to 81 more) | ⨁⨁⨁⨁  High |
| **Polyp detection rate** | | | | | | | | | | | |
| 9 | randomised trials | not serious | not serious | not serious | not serious | publication bias strongly suspected^a^ | 539/1124 (48.0%) | 467/1169 (39.9%) | **RR 1.16**  (1.06 to 1.26) | **64 more per 1,000**  (from 24 more to 104 more) | ⨁⨁⨁◯  Moderate |
| **Boston Bowel Preparation Scale Score** | | | | | | | | | | | |
| 13 | randomised trials | not serious | serious^b^ | not serious | not serious | publication bias strongly suspected^c^ | 1788 | 1779 | - | MD **0.34 higher**  (0.14 higher to 0.54 higher) | ⨁⨁◯◯  Low |
| **Ottawa Bowel Preparation Scale Score** | | | | | | | | | | | |
| 3 | randomised trials | not serious | serious^b^ | not serious | not serious | none | 572 | 370 | - | MD **0.98 lower**  (2.55 lower to 0.59 higher) | ⨁⨁⨁◯  Moderate |
| **Cecal Insertion Time** | | | | | | | | | | | |
| 8 | randomised trials | not serious | not serious | not serious | serious^d^ | publication bias strongly suspected^e^ | 1346 | 1392 | - | MD **0.13 lower**  (0.48 lower to 0.21 higher) | ⨁⨁◯◯  Low |
| **Cecal Intubation Rate** | | | | | | | | | | | |
| 15 | randomised trials | not serious | not serious | not serious | not serious | none | 2340/2376 (98.5%) | 2278/2316 (98.4%) | **RR 1**  (1 to 1) | **0 fewer per 1,000**  (from 0 fewer to 0 fewer) | ⨁⨁⨁⨁  High |

**CI:** confidence interval; **MD:** mean difference; **RR:** risk ratio

Explanations

a. Major asymmetry

b. High heterogeneity and p < 0.05; some 95% confidence intervals do not overlap; variation in effect

c. Egger p-value < 0.05

d. Variation in effect

e. Minor asymmetry

**Supplementary Figure S1:** Traffic Light Plot showing Risk of Bias Assessment


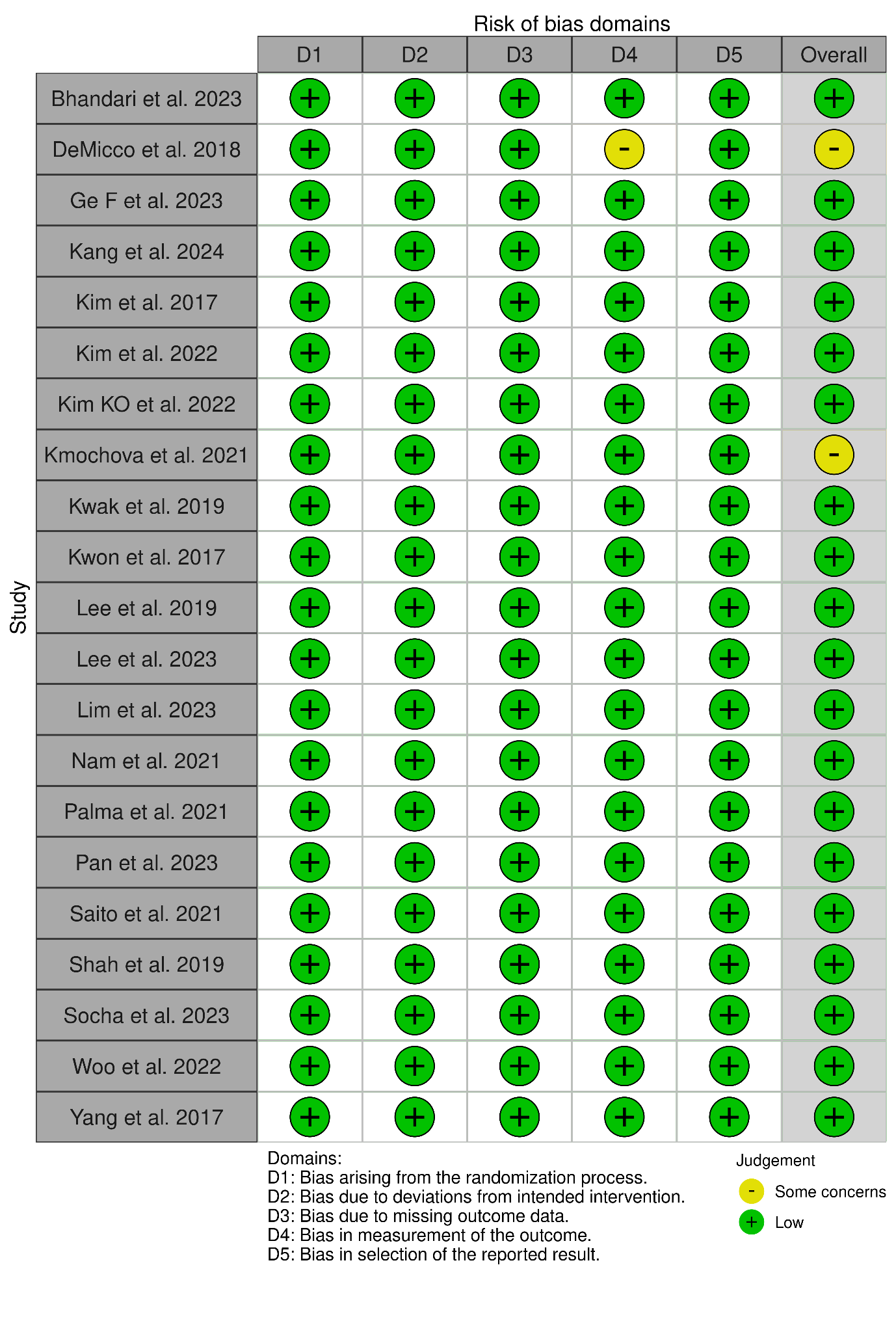


**Supplementary Figure S2:** Summary Plot showing Risk of Bias Assessment


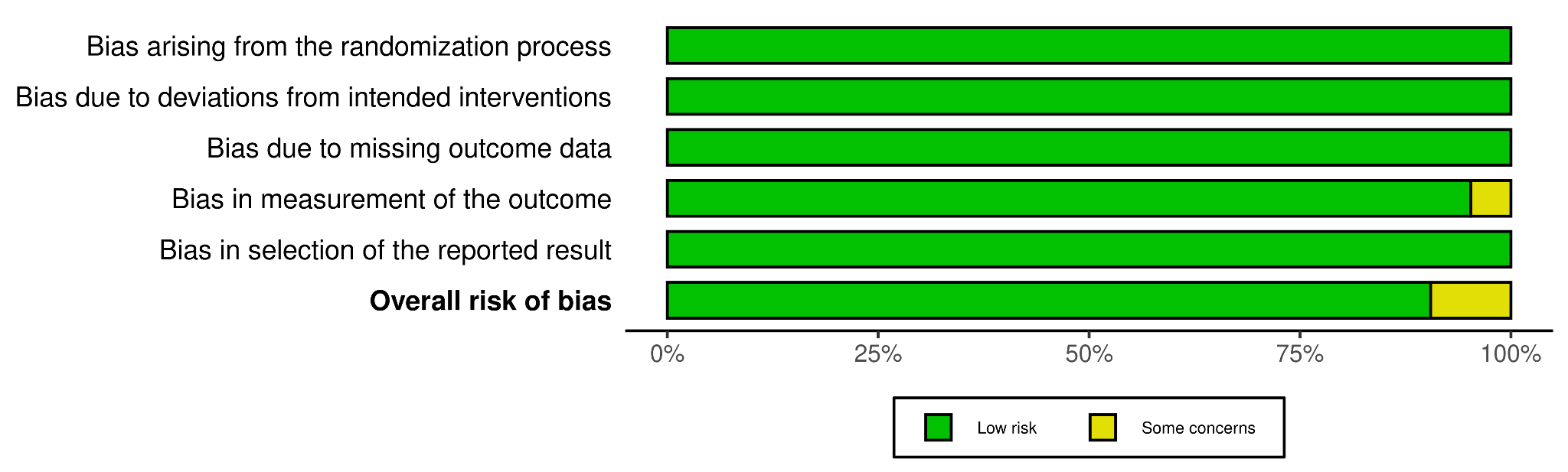


**Supplementary Figure S3:** L1O Analysis for BBPS Score Morning Colonoscopy Subgroup


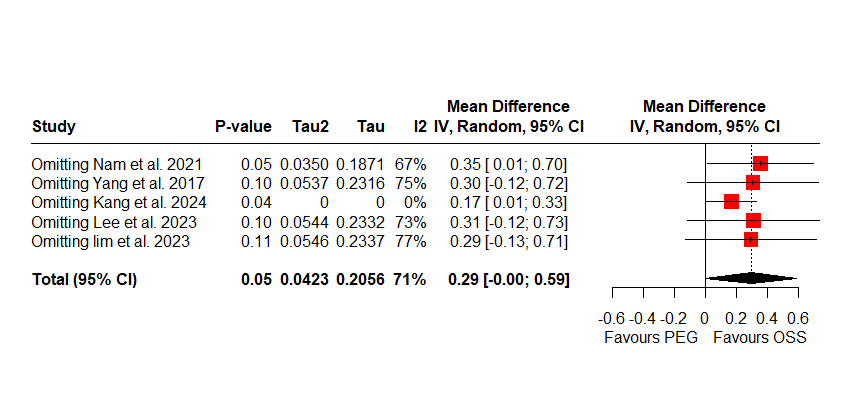


**Supplementary Figure S4:** L1O Analysis for BBPS Score Outpatient Clinical Setting Subgroup

**
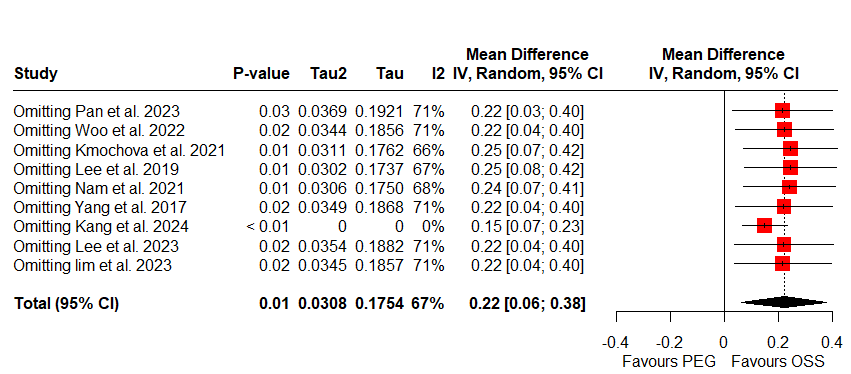
**

**Supplementary Figure S5:** L1O Analysis for BBPS Score BMI < 25 kg/m2 Subgroup

**
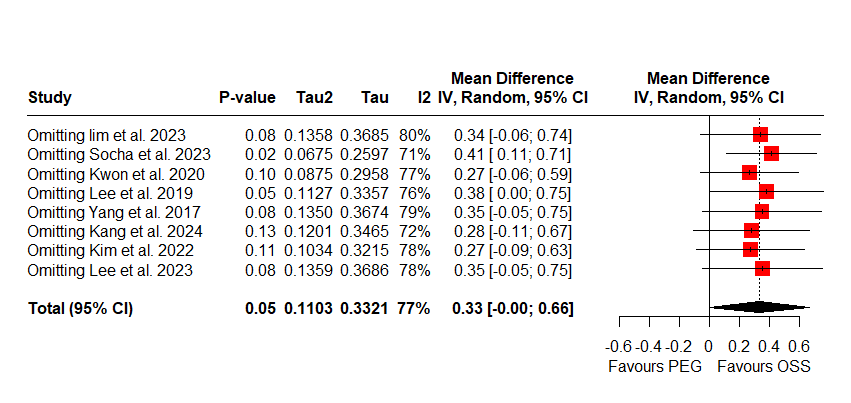
**

**Supplementary Figure S6:** L1O Analysis for Cecal Insertion Time


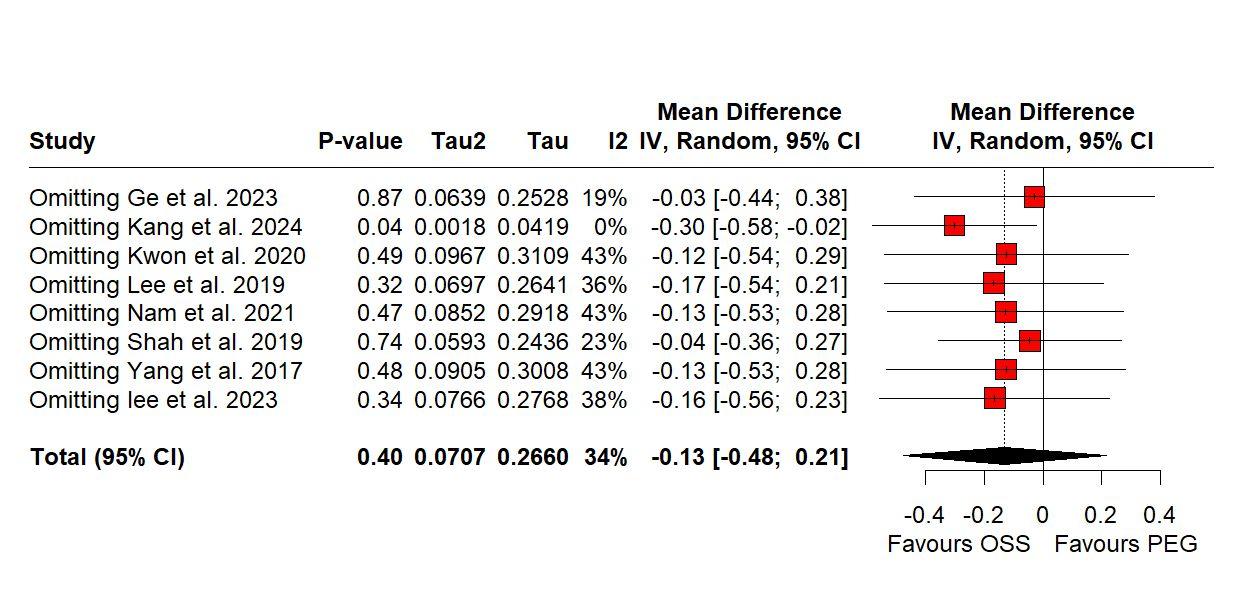


**Supplementary Figure S7:** Subgroup Analysis of Adenoma Detection Rate (ADR) on basis of Asian Studies


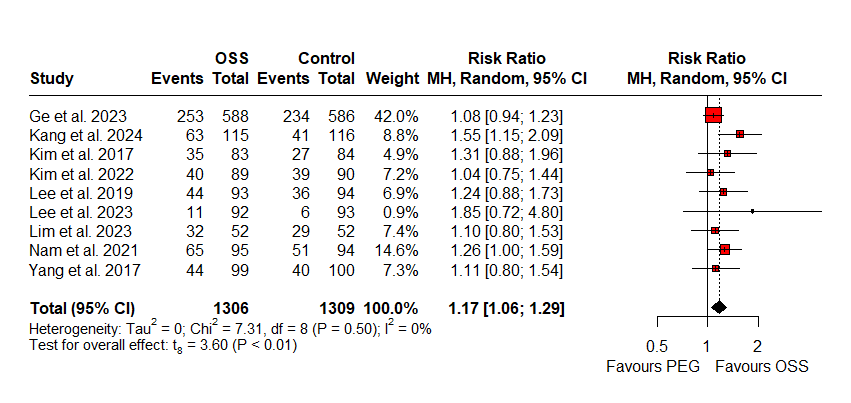


**Supplementary Figure S8:** Subgroup Analysis of Polyp Detection Rate (PDR) on basis of Asian Studies


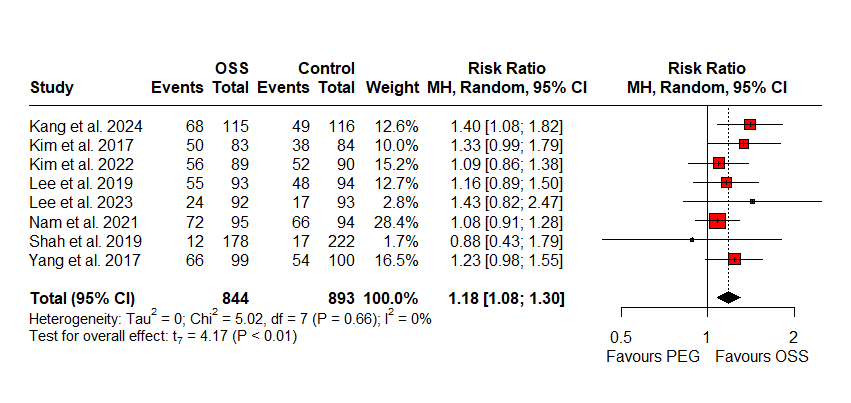


**Supplementary Figure S9:** Subgroup Analysis of BBPS score on basis of Asian Studies


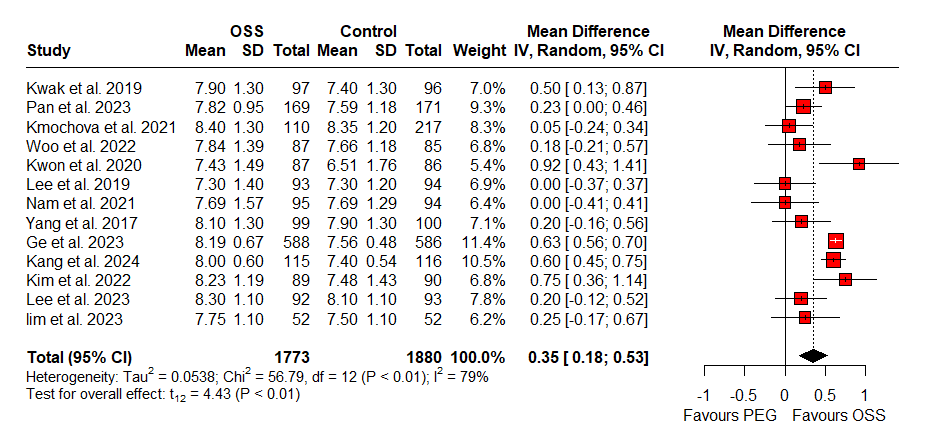

Supplement: Supplementary file 1 — Table S1: Detailed Search Strategy of Each Database Table S2: GRADE Certainty of Evidence Assessment Figure S1: Traffic Light Plot showing Risk of Bias Assessment Figure S2: Summary Plot showing Risk of Bias Assessment Figure S3: L1O Analysis for BBPS Score Morning Colonoscopy Subgroup Figure S4: L1O Analysis for BBPS Score Outpatient Clinical Setting Subgroup Figure S5: L1O Analysis for BBPS Score BMI < 25 kg/m2 Subgroup Figure S6: L1O Analysis for Cecal Insertion Time Figure S7: Subgroup Analysis of Adenoma Detection Rate (ADR) on basis of Asian Studies Figure S8: Subgroup Analysis of Polyp Detection Rate (PDR) on basis of Asian Studies Figure S9: Subgroup Analysis of BBPS score on basis of Asian Studies [file DEO2-5-e70113-s001.docx]
